# Inhibition of Myostatin Reduces Collagen Deposition in a Mouse Model of Oculopharyngeal Muscular Dystrophy (OPMD) With Established Disease

**DOI:** 10.3389/fphys.2020.00184

**Published:** 2020-03-05

**Authors:** Pradeep Harish, Leysa Forrest, Shanti Herath, George Dickson, Alberto Malerba, Linda Popplewell

**Affiliations:** Department of Biological Sciences, Centre of Gene and Cell Therapy and Biomedical Sciences, Royal Holloway, University of London, Egham, United Kingdom

**Keywords:** OPMD, myostatin, antibody, RK35, muscular dystrophy

## Abstract

**Background:**

Oculopharyngeal muscular dystrophy (OPMD) is a late-onset muscle disease presented by ptosis, dysphagia, and limb weakness. Affected muscles display increased fibrosis and atrophy, with characteristic inclusion bodies in the nucleus. Myostatin is a negative regulator of muscle mass, and inhibition of myostatin has been demonstrated to improve symptoms in models of muscular dystrophy.

**Methods:**

We systemically administered a monoclonal antibody to block myostatin in the A17 mouse model of OPMD at 42 weeks of age. The mice were administered a weekly dose of 10 mg/kg RK35 intraperitonially for 10 weeks, following which serum and histological analyses were performed on muscle samples.

**Results:**

The administration of the antibody resulted in a significant decrease in serum myostatin and collagen deposition in muscles. However, minimal effects on body mass, muscle mass and myofiber diameter, or the density of intranuclear inclusions (INIs) (a hallmark of disease progression of OPMD) were observed.

**Conclusion:**

This study demonstrates that inhibition of myostatin does not revert muscle atrophy in a mouse model with established OPMD disease, but is effective at reducing observed histological markers of fibrosis in the treated muscles.

## Introduction

Oculopharyngeal muscular dystrophy (OPMD) is a late onset muscle wasting disease, whose clinical diagnosis is often compounded by the onset of sarcopenia associated muscle loss (Tome et al., 1997; Blumen et al., 2000; Trollet et al., 2010). The primary clinical indications for the disease involves ptosis, dysphagia, and proximal limb weakness. The genetic basis of the disease revolves around a mutation in the *PABPN1* gene whose product regulates poly (A) tail length on mRNAs, controls the use of alternative polyadenylation (APA) sites, and influences pre-mRNA splicing among other roles (Harish et al., 2015). In OPMD, mutated PABPN1 has a poly-alanine expansion at the N terminus of the protein, resulting in 11–18 repeats instead of the normal 10 present in unaffected individuals (Brais et al., 1998; Blumen et al., 2000). The alanine expansion results in protein misfolding and consequent accumulation in the nuclei as intranuclear inclusion bodies (INI) (Harish et al., 2018). These INI bodies also sequester other molecules such as poly(A)-containing RNA, various transcription factors of the proteasome ubiquitin pathway (ubiquitin and 20S catalytic proteasomal subunit), molecular chaperones (HDJ-1, HSP70), heterogeneous nuclear ribonucleoprotein A1 (HNRPA1) and arginine methyltransferares (Harish et al., 2018). The sequestration of these proteins may induce defects in transcriptomic or protein folding pathways (Tavanez et al., 2009; Malerba et al., 2017). Current methods to ameliorate disease symptoms are surgical in nature, however, various small molecule and gene therapy strategies have been proposed that directly or indirectly target the INI bodies (Harish et al., 2018). Concordant with other muscular dystrophies, moderate muscle atrophy (especially in non-somitically derived muscles) has also been described in patients with OPMD (Schmitt and Krause, 1981; Little and Perl, 1982), and hence therapeutic agents that target muscle mass may ameliorate symptoms in this disease state.

Myostatin is a known regulator of muscle mass and has been examined as a therapeutic target to ameliorate symptoms of dystrophy, cachexia, and sarcopenia (Rodgers and Garikipati, 2008; Sartori et al., 2013; Mouisel et al., 2014). While primary myostatin signaling is effected as a balance between the bone morphogenetic protein (BMP) and activing receptor IIB (ACTRIIB) signaling pathways, secondary signaling mechanisms also influence cell growth via interactions with the IGF-1, p21/Cdk, Wnt signaling pathways (Rodgers and Garikipati, 2008; McPherron, 2010; Sartori et al., 2013). Studies in myostatin null mice report an increased bone mineral density (as compared to wild-type controls) and ejection fraction, resistance to diet induced obesity, dyslipidemia, atherogenesis, hepatic steatosis and macrophage infiltration, besides a substantial improvement in muscle mass (White and LeBrasseur, 2014). Inhibition of myostatin on disease progression has been studied in aged *mdx* mice (modeling Duchenne muscular dystrophy) and C57 (wildtype) model systems utilizing various strategies, and report variable levels of efficacy (LeBrasseur et al., 2009; Murphy et al., 2010; Arounleut et al., 2013). Unsurprisingly, a variety of strategies to disrupt myostatin signaling are in pre-clinical and clinical development, including but not limited to propeptide, gene therapy, gene editing, ligand traps, and monoclonal antibodies (Wagner et al., 2002, 2008; Bogdanovich et al., 2005; Mendell et al., 2015; Bhattacharya et al., 2017; Campbell et al., 2017).

We recently published that immunological blockade of myostatin in young (12 week old) A17 mice improved muscle mass, muscle force, and reduced collagen deposition (Harish et al., 2019). The A17 model mouse, which expresses a bovine exp*PABPN1* transgene (17 expanded alanine residues driven by a human alpha actin promoter), has been used routinely in various studies as it models OPMD disease progression on a cellular level (Trollet et al., 2010).

In this current study, we utilize the same treatment regimen in relatively older OPMD mice to assess the impact of myostatin inhibition in models with pre-existing muscle atrophy. Here, we show that administration of the anti-myostatin monoclonal antibody RK35 to 42 week old A17 mice for a period of 10 weeks, while not affecting accumulation of intranuclear aggregates, or loss of body mass, muscle atrophy, or muscle strength, does, however, reduce deposition of fibrotic collagen proteins. This would suggest the proposed anti-myostatin therapy may have a role as an adjuvant treatment option in established disease states in addition to gene therapy or small molecule strategies to ameliorate disease symptoms and presents future avenues to be investigated (Malerba et al., 2017, 2019a).

## Materials and Methods

### Animal Handling

A17 and FvB mice were bred in-house and all mice were housed individually with food and water *ad libitum* in a minimal disease facility at Royal Holloway, University of London (Davies et al., 2005). Individual mice were identified by ear-notching at about 4 weeks of age. Due to the heterozygous nature of the disease model, the OPMD mice were analyzed to confirm the genotype by PCR as described previously (Trollet et al., 2010). 42 week old male mice were weighed prior to each injection, at the same time of day. Initial body weights were used to evenly distribute animals among cohorts to ensure equivalent average body weights prior to the commencement of experimental protocols. In this experiment, we administered the anti-myostatin blocking antibody RK35 (Pfizer, United States) diluted in sterile saline (Sigma-Aldrich, United Kingdom) for a final volume of 200 μl. The antibody was injected weekly at 10 mg/kg i.p. for 10 weeks into A17 and FvB mice [9 A17 mice treated with the anti-myostatin antibody RK35 (A17 + RK35), 8 A17 mice treated with saline as vehicle control (A17 + saline), 10 FvB mice treated with the antibody RK35 (FvB + RK35), and 10 FvB mice treated with saline as a strain control (FvB + saline)]. *In vivo* experiments were conducted under statutory Home Office recommendation; regulatory, ethics, and licensing procedures and the Animals (Scientific Procedures) Act 1986 (Project License 36A9994E).

### Sample Collection, Processing and Immunological Detection of Serum Myostatin

Mice were euthanized 1 week after the last injection of RK35 at the 53rd week of age, and the Tibialis Anterior (TA) muscle was harvested, weighed and mounted in O.C.T. compound (Thermo Fisher Scientific, United Kingdom), frozen in 2-methylbutane (isopentane) chilled with liquid nitrogen, and stored at −80°C. Blood was extracted by cardiac puncture, allowed to clot overnight at 4°C, serum extracted and spun down successively with increasing speeds at 1000 g, 2000 g, and 3500 g to remove residual cells. The serum sample thus collected was stored at −80°C. Serum samples were then subject to activation (R&D Systems, United States, DY010) and sandwich ELISA (R&D Systems, United States, DGDF80) to detect serum myostatin as per manufacturer’s recommended protocol. Additional samples from mice subject to the same treatment regimen from 12 weeks of age obtained via a previously conducted experiment were also used in this study (Harish et al., 2019).

### Histological and Immunohistochemical Analysis

Transverse sections of the tissue were sectioned at 10–12 different intervals at a thickness of 10 μm, along the length of the muscle, allowing the maximal cross-sectional area (CSA) to be determined, the sections were mounted on coated slides (VWR International, United Kingdom), and stored at −80°C. Sections were then air-dried, fixed, stained with anti-PABPN1 (rabbit monoclonal, diluted 1:100, Abcam ab75855, overnight at 4°C), anti-laminin (rat monoclonal, diluted 1:800, Sigma-Aldrich L0663, 1 h at room temperature) and anti- collagen VI (rabbit polyclonal, Abcam ab6588, 1:200, 1 h room temperature) antibodies using previously established protocols (Malerba et al., 2017). Slides were stained with DAPI to visualize nuclei and coverslips were mounted using mounting medium (Vector Labs, United States). Whole muscle images (for laminin based fiber morphometry) or random fields (for PABPN1 and collagen immunostaining) were captured using a microscope (Zeiss, United Kingdom). For analysis of fiber morphometry, the median minimal feret’s diameter from 1000 randomly selected fibers were determined for TA muscles for each individual animal analyzed (Harish et al., 2019; Malerba et al., 2019a). For picrosirius red staining, sections were air dried, fixed in PFA 4%, and stained in a 0.3% solution of Sirius red in saturated aqueous picric acid, followed by a treatment with 0.5% acetic acid. Imaging of sirius red staining was performed under white light to analyze collagen deposition.

### Statistical Analyses

After checking for the conditions of normality and homoscedasticity, a one way ANOVA was performed to compare multiple groups. Multiple comparison were performed with Bonferroni correction to correct for multiple testing used. All statistical techniques were performed using GraphPad Prism v7.00 (GraphPad Software, California, United States).

## Results

### Treatment With Anti-myostatin Antibody Has Minimal Effect on Body Mass in 52-Week Old OPMD Mice

**FIGURE 1:**
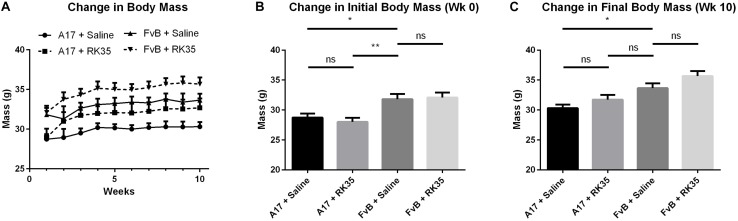
Treatment with RK35 antibody does not affect body mass in 52 week old A17 mice: Mice (*n* = 8–10) were weighed and administered a weekly regimen of either saline or the RK35 antibody (10 mg/kg i.p.) for 10 weeks from the 42nd week of age. **(A)** The average body mass per group plotted against weeks shows that body masses **(B)** are significantly different at the beginning of the experiment and **(C)** the difference is maintained after 10 weeks of treatment. Data presented as mean with SEM, with *p*-values obtained by ANOVA after a Bonferroni correction (*p < 0.05; **p < 0.01).

To monitor the effects of inhibiting myostatin in a mouse model of OPMD, where the disease is already established, 8–10 male A17 and FvB mice at 42 weeks of age were weighed and the anti-myostatin antibody RK35 (10 mg/kg IP) or an equivalent volume of saline was administered weekly for 10 weeks (Figure 1A).

At the beginning of the study a 10% (*p* < 0.05) difference in the initial body mass existed between A17 mice and FvB mice (Figure 1B). This difference was maintained throughout the study. The treatment of A17 mice with the antibody caused a non-significant increase in final body mass of 5% (*p* > 0.05) when compared to the saline treated A17 mice (Figure 1C). The treatment of FvB control mice resulted in no significant changes in body mass observed.

### Treatment With Anti-myostatin Antibody Has Minimal Effect on Muscle Mass and Muscle Fiber Diameter, and Intranuclear Inclusion Density in 52-Week Old OPMD Mice

**FIGURE 2:**
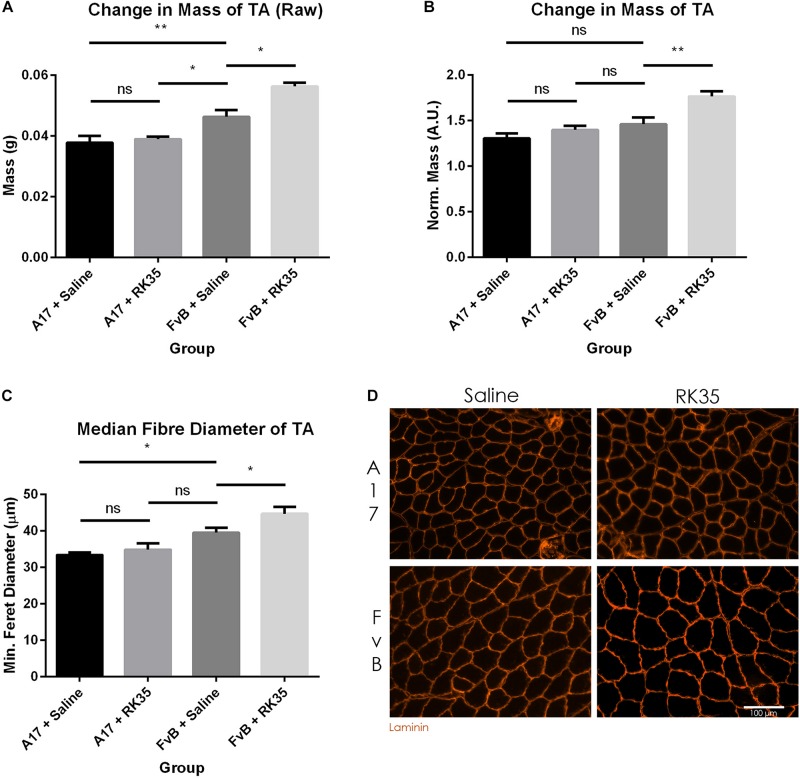
Treatment with RK35 antibody has does not affect muscle mass and myofiber diameter in OPMD mice: Mice were administered with a weekly regimen of either saline or the RK35 antibody (10 mg/kg i.p.) for 10 weeks from 42-weeks of age. The TA was sampled and **(A)** raw muscle mass or **(B)** muscle mass normalized to the initial body mass is presented. Next, muscle samples from five randomly selected mice per group were sectioned and immunostained for laminin. **(C)** 1000 fibers were randomly selected for the TA, and the median minimum feret diameter was analyzed. **(D)** Representative images used to generate myofiber diameter data are shown on the bottom right panel with the scale bar representing 100 μm. The FvB mice treated with the antibody consistently displayed an increased muscle mass and myofiber diameter, with no changes observed in the A17 mice. The average of median minimum feret diameter per group was plotted, Data presented as mean with SEM, with *p*-values obtained by ANOVA after a Bonferroni correction (*p < 0.05; **p < 0.01).

When examining the change in muscle mass, we detected a significant increase of 20% (*p* < 0.01) in raw muscle masses of A17 and FvB muscles treated with saline (Figure 2A). No difference in the mass of the TA (normalized to the initial body mass) was found between the untreated A17 and FvB mice (Figure 2B). It should be noted that the administration of the antibody RK35 resulted in no changes to either the normalized or raw muscle mass of the TA in the A17 mice when compared to saline controls. However, a significant increase in muscle mass in the treated FvB controls [19% (*p* < 0.05) increase in normalized and raw muscle masses] was observed when compared to the untreated FvB mice, demonstrating the functionality of the administered antibody and suggesting that this treatment was not able to reverse established muscle atrophy (Figures 2A,B).

To better characterize the muscle atrophy in aged A17 mice, we analyzed the myofiber size. The decrease in muscle mass in the A17 mice was accompanied by a reduction of myofiber diameter in the TA by 18% (*p* < 0.05) when compared to FvB mice (Figures 2C,D). The administration of RK35 to A17 mice resulted in a minimal increase in myofiber diameter of the TA (Figure 2C). In accordance with the increase in muscle mass observed in FvB mice, the myofiber diameter in TA muscles of age-matched FvB controls increased by 13% (*p* < 0.05) compared to those of saline-treated FvB mice (Figure 2C). Finally, the administration of the RK35 antibody did not affect the levels of INIs in the TA in this study (Supplementary Figure S1).

### Anti-myostatin Antibody Significantly Reduces Collagen Deposition in A17 Mice

**FIGURE 3:**
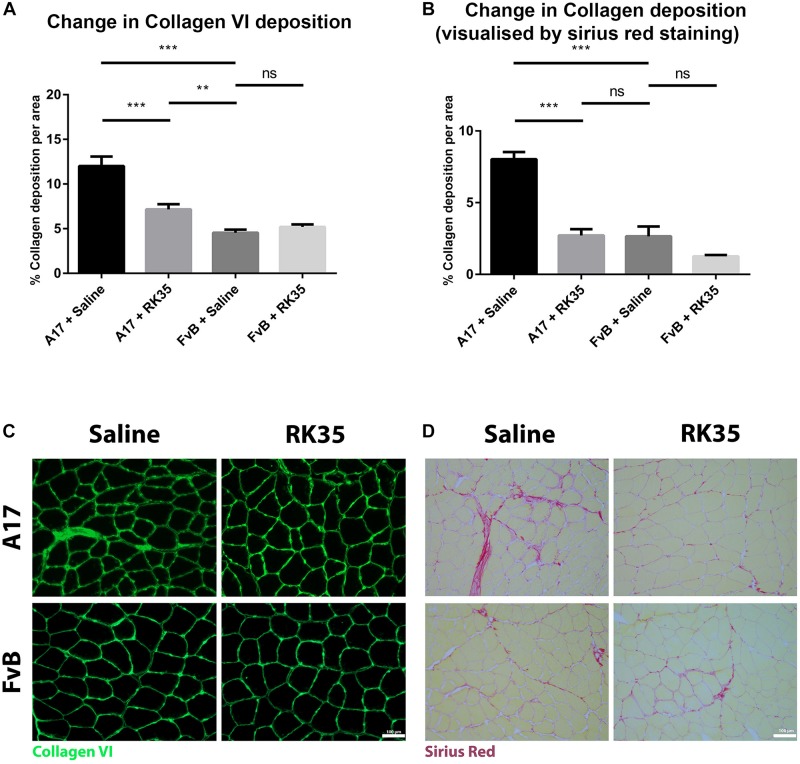
Treatment with RK35 antibody reduces collagen deposition in OPMD mice: Mice were administered with a weekly regimen of either saline or the RK35 antibody (10 mg/kg i.p.) for 10 weeks from 42-weeks of age. Five randomly selected whole TA muscle sections from all groups were stained for **(A)** collagen VI and **(B)** picrosirius red, and five random fields were imaged and analyzed for the percentage area of collagen staining. **(C,D)** Representative images used to generate data are shown below the respective graphs, with the scale bar representing 100 μm. The mice treated with the antibody consistently displayed a reduced collagen deposition, with the area of collagen in the disease model normalized to wild-type levels. The average area per group is plotted, bars representing SEM, with *p*-values obtained by ANOVA after a Bonferroni correction (**p < 0.01; ***p < 0.001).

We next examined the effect of inhibition of myostatin on the deposition of collagen proteins as histopathological markers of fibrotic tissue deposition in the muscles. Untreated A17 mice displayed a significantly increased collagen deposition when compared to control FvB mice [with the control mice displaying 60% (*p* < 0.05) less collagen deposition than the A17 mice] (Figures 3A,C). Inhibition of myostatin by administered RK35 antibody reduced deposition of collagen VI in the TA muscle of A17 mice by 40% (*p* < 0.001) and general collagen deposition as visualized by Sirius red staining by 29% (*p* < 0.01) when compared to untreated A17 mice (Figures 3B,D). This reduction in collagen deposition (as determined by Sirius red staining) in treated A17 mice was particularly marked since they were ameliorated to the levels seen in the FvB controls (4% difference, *p* > 0.05) (Figure 3B).

### Treatment With Anti-myostatin Antibody Significantly Reduces Serum Myostatin 52-Week Old OPMD Mice

We next performed a sandwich ELISA in order to quantify the efficacy of the anti-myostatin antibody RK35 used in this study to reduce the levels of serum myostatin. To establish any age-dependency of response, we utilized serum samples from 52 week old or 12 week old A17 and FvB mice treated systemically with either saline or RK35 for 10 weeks, at 10 mg/kg i.p. (*n* = 5 per group) obtained from a previous study (Harish et al., 2019).

**FIGURE 4:**
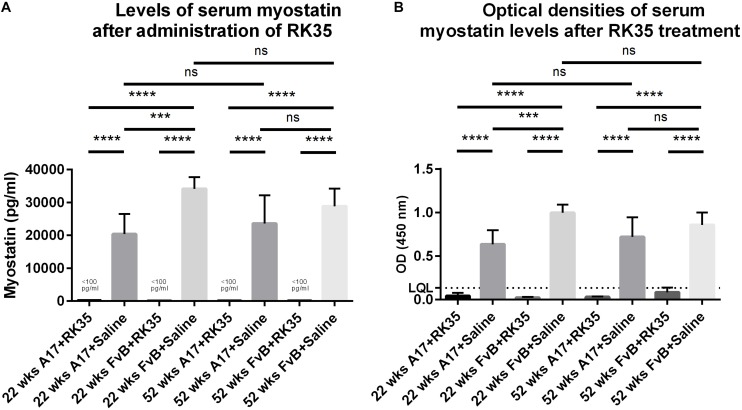
Treatment with RK35 significantly reduces levels of serum myostatin: Serum samples from mice (*n* = 5) were subject to a weekly regimen of either saline or the anti-myostatin RK35 antibody i.p. for 10 weeks from 42 weeks of age, or samples were utilized from a previously published study (Harish et al., 2019) were subject to a sandwich ELISA to detect myostatin. **(A)** The administration of the antibody resulted in the reduction of serum myostatin below the lower quantitation limit (LQL) of 100 pg/ml. **(B)** Optical densities corrected for background readings are plotted for each group analyzed. Data presented as mean with SEM, with *p*-values obtained by ANOVA after a Bonferroni correction (***p < 0.001, ****p < 0.0001).

When considering the control samples from the 22 week old mice, the levels of serum myostatin are 40% higher (*p* < 0.01) in the FvB mice than the A17 mice (Figure 4A). Effective reduction of myostatin using the RK35 antibody levels below the detectable limits of the ELISA is evident for both A17 and FvB young mice (Figure 4B). For the older mice, while no significant difference was observed in serum myostatin levels between 52 week old A17 and FvB mice, the treatment with the anti-myostatin antibody RK35 again resulted in reduction of serum myostatin to below detectable levels for both strains of mice (Figure 4A). When comparing the levels of serum myostatin between the two age groups, no significant difference was observed between untreated A17 mice at 22 weeks of age or 52 weeks of age. Similarly, no significant difference was observed between untreated FvB mice at 22 weeks of age or 52 weeks of age (Figure 4A).

## Discussion

Myostatin is a negative regulator of muscle mass (Rodgers and Garikipati, 2008). Subsequently, the most striking result of inhibition of myostatin involves significant increases in muscle mass and muscle fiber diameter (McPherron et al., 1997). Hence, agents that either directly or indirectly disrupt myostatin signaling are being investigated by various pharmaceutical industries as a generic broad-spectrum therapeutic strategy to alleviate muscle loss arising from cachexia, sarcopenia, or muscular dystrophy (Rodgers and Garikipati, 2008; Lu-Nguyen et al., 2015, 2017; Camporez et al., 2016; Andre et al., 2017; Tinklenberg et al., 2017). OPMD is a rare poly-alanine expansion induced protein aggregopathy, and has been modeled in the A17 mouse strain (Davies et al., 2005). It is reported that the disease progression manifests as visible changes in muscle mass at 18 weeks of age in the model mice (Trollet et al., 2010). Recent efforts to ameliorate symptoms have focused on small molecule approaches to target protein aggregation and gene therapy approaches to correct the mutation (Malerba et al., 2017, 2019a,b). In our previous study we showed that the administration of the RK35 antibody counteracted the muscle atrophy and improved histopathological features of the disease (Harish et al., 2019). Screening of therapeutic reagents in relatively younger murine models of late-onset diseases, such as OPMD, presents challenges in translating into clinical trials (Harish et al., 2015). Furthermore, pathogenesis of disease in OPMD mice is markedly different from disease progression in human patients. While the disease profile in the A17 mouse model is present in all skeletal muscles, the etiological profile in humans is localized to the extraocular and pharyngeal muscles, with proximal limb muscle involvement depending on disease progression and severity (Davies et al., 2005; Trollet et al., 2010). Therefore, the rationale behind this study was to assess the efficacy of myostatin inhibition in an OPMD disease model with an established disease state.

The A17 mouse model at 52-weeks of age possesses a lower body and muscle mass than the FvB controls, along with a significantly reduced myofiber diameter and increased collagen deposition. The inhibition of myostatin produced no increase in muscle mass in the A17 model mice, which is in contrast to what we observed in younger A17 mice (Harish et al., 2019). Interestingly, the functionality of the antibody was evident in the healthy 52-week old FvB control mice, with significantly increased muscle mass and myofiber diameters observed in this study. We originally hypothesized that different levels of serum myostatin between A17 and FvB mice could be implicated in the differing efficacy of myostatin inhibition observed between the two age groups. Indeed, Mariot et al. (2017) have reported that levels of serum myostatin might be directly dependant on type of disease and state of disease progression. We report here that the levels of serum myostatin are significantly lower in 22-week old A17 mice when compared to the FvB controls and this correlates well with the results obtained in younger mice (Harish et al., 2019). However, no such difference exists in the 52-weeks cohort.

The mechanistic reason behind this differing effect will need to be investigated further in a future study. We speculate a combined sarcopenic and dystrophic phenotype may irreversibly alter muscle structure and attenuate the efficacy of the therapeutic regimen as compared to models in earlier stages of the disease, thereby preventing regrowth of the muscle mass with myostatin inhibition. For example, we speculate crucial molecules involved in the myostatin pathway (e.g., the activin receptor IIb) or molecular pathways affecting the muscle growth (e.g., Insulin like growth factors) may be dysregulated as result of the OPMD pathology significantly reducing the impact of myostatin inhibition. Alternatively, other molecules which negatively regulate muscle mass (such as activin A), might reduce the efficacy of myostatin knockdown in this older cohort (Latres et al., 2017).

Promisingly, while the untreated A17 mice displayed a marked increased collagen deposition when compared to the untreated FvB controls in the TA, the administration of the treatment regimen reduced the collagen deposition. Pathophysiological fibrosis associated with aging/dystrophy results in sarcolemmal fragility, disturbances in calcium ion homeostasis, inflammation, reduction of motile and contractile functions, and reduction of available tissue for therapeutic intervention (Desguerre et al., 2009; Mann et al., 2011). Attenuation of muscle fibrotic processes may have long term benefits to a dystrophic muscle in terms of promoting regeneration, muscle force transfer, and maintaining structural integrity. Myostatin is a known regulator of fibroblast activation and apoptosis (Li et al., 2008), with anti-myostatin agents shown to have an anti-fibrotic effect in other models of muscular dystrophy (Wagner et al., 2002; Li et al., 2012).

It is interesting to note in our studies that while inhibiting myostatin in younger mice improved muscle atrophy and reduced collagen deposition (Harish et al., 2019), the inhibition of myostatin in older mice had only a significant effect on reducing collagen deposition. This underlines the importance of elucidating the mechanism of myostatin inhibition on pre-existing muscle atrophy, as this may have an impact on translating anti-myostatin treatment regimens to the clinic. Indeed, most direct immunological blockers of myostatin activity have had limited success in clinical trials (Wagner et al., 2008; Campbell et al., 2017), with primary endpoints being a readout of lean mass, muscle volume, inflammatory responses or muscle regeneration. It may be informative to verify the therapeutic benefit of the anti-fibrotic action of myostatin inhibition in late-onset muscle diseases.

## Data Availability Statement

All datasets generated for this study are included in the article/Supplementary Material.

## Ethics Statement

The animal study was reviewed, approved, and performed under regulations and recommendations laid down by the UK Home Office (ASPA 1986).

## Author Contributions

PH, AM, LP, and GD contributed to the study design. PH, AM, SH, and LF participated in the data collection and interpretation. PH and AM prepared the manuscript. All authors provided the manuscript review and revisions.

## Conflict of Interest

The authors declare that the research was conducted in the absence of any commercial or financial relationships that could be construed as a potential conflict of interest.

## Supplementary Material

FIGURE S1 Treatment with RK35 does not affect the amount of intranuclear aggregates: Mice were subject to a weekly regimen of either saline or the anti-myostatin RK35 antibody i.p. for 10 weeks from 42 weeks of age. **(A)** Five muscle samples from all groups were stained for endogenous PABPN1 after a 1M KCl treatment with the scale bar representing 50 μm, and five random fields were imaged and analyzed for the percentage number of positive PABPN1 stains to all myonuclei. **(B)** The administration of the treatment regimen did not change the number of intranuclear aggregates observed. The average density is plotted, bars representing SEM, with *p*-values obtained by ANOVA after a Bonferroni correction (****P* < 0.001).
