# Supplementary figures and images for: Inhibition of Myostatin Reduces Collagen Deposition in a Mouse Model of Oculopharyngeal Muscular Dystrophy (OPMD) With Established Disease
Source: Front Physiol. 2020 Mar 5;11:184. doi: 10.3389/fphys.2020.00184 (PMC7066371; doi:10.3389/fphys.2020.00184)

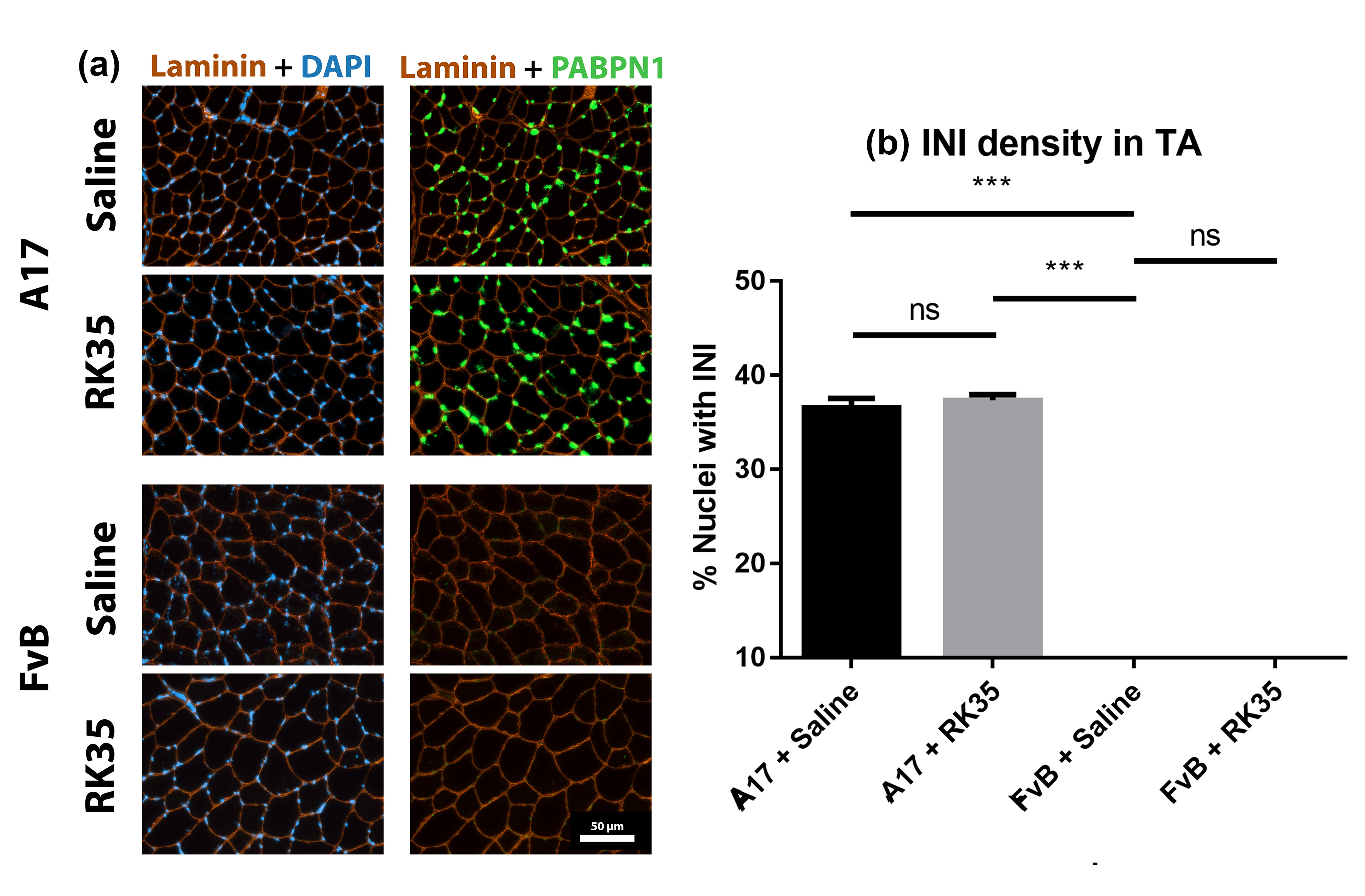

Supplement: FIGURE S1 — Treatment with RK35 does not affect the amount of intranuclear aggregates: Mice were subject to a weekly regimen of either saline or the anti-myostatin RK35 antibody i.p. for 10 weeks from 42 weeks of age. (A) Five muscle samples from all groups were stained for endogenous PABPN1 after a 1M KCl treatment with the scale bar representing 50 μm, and five random fields were imaged and analyzed for the percentage number of positive PABPN1 stains to all myonuclei. (B) The administration of the treatment regimen did not change the number of intranuclear aggregates observed. The average density is plotted, bars representing SEM, with p-values obtained by ANOVA after a Bonferroni correction (***P < 0.001). [file Image_1.JPEG]
